# Cognitive Impairment and Dementia Prevalence in the Northern Region of Peru: An Epidemiological Overview

**DOI:** 10.1002/alz.092445

**Published:** 2025-01-09

**Authors:** Jonathan Adrian Zegarra‐Valdivia, Leandro Aron Perez‐Fernandez, Milagros del Rocio Casimiro‐Arana, Harold Alessandro Arana‐Nombera, Viviana Nayelli Gallegos‐Manayay, Reyna Elizabeth Alamo‐Medina, María del Rosario Oliva‐Piscoya, Eduardo Sebastián Abanto‐Saldaña, Maria Celinda Cruz Ordinola, Nobuko Vásquez‐Zuñe, Sheila Castro‐Suarez, Carmen Paredes Manrique, Lisseth Catherime Detquizan Pérez, Ernesto Sebastian Lozano Fernández, Leslie Magdalena Lozada Tantarico, Zaraíd Maria Chancafe Neciosup, Mariana Villegas, Julio Inga, Sintia Vásquez, Karolay Lluén Navarrete, Erick Muñoz, Nilson Domínguez, Julio Ramírez, Nataly Diaz, Nelson Salinas, Johnny Ramos, Patricia Ventura, Deliz Liza Serrán, Josetty Coronado

**Affiliations:** ^1^ Universidad Señor de Sipán, Chiclayo Peru; ^2^ Universidad San Martín de Porres, Chiclayo Peru; ^3^ Global Brain Health Institute (GBHI), University of California San Francisco, San Francisco, CA USA; ^4^ Instituto Nacional de Ciencias Neurologicas, Lima Peru; ^5^ Universidad Tecnológica del Perú, Arequipa Peru

## Abstract

**Background:**

The prevalence of dementia in Peru’s northern regions is poorly documented, largely due to the scarcity of studies employing validated assessment tools for the elderly. Notably, this area is marked by pronounced disparities, encompassing a wide range of socioeconomic statuses and predominantly low educational attainment. The confluence of risk factors, including educational and socioeconomic deprivation, prevalent diseases, suboptimal health conditions, chronic stress, and lifelong malnutrition, poses a significant risk of escalating dementia cases over the next two decades.

**Objective:**

This study aims to ascertain the prevalence of cognitive impairment among a socioeconomically diverse cohort of individuals aged 50 and above in Chiclayo, attending various community settings such as Senior Centers, Nursing Homes, and neighborhood groups. It zeroes in on the northern region, notorious for its pronounced cognitive disparities largely attributed to educational variances.

**Methods:**

Employing a descriptive epidemiological approach with a cross‐sectional design, this study utilizes a two‐stage cluster sampling methodology. To date, over 400 participants from the northern region have been evaluated. The assessment includes cognitive screenings to pinpoint cognitive deficits and functionality issues, alongside the exclusion of potential emotional disorders.

**Results:**

Initial findings from 99 qualifying participants reveal the Rowland Universal Dementia Assessment Scale ‐ Peru (RUDAS‐P) as an effective tool for identifying cognitive impairment and dementia in populations with limited education. Despite varied educational backgrounds, 25% exhibited cognitive impairment, and 16% showed dementia signs, aligning with findings from other national studies. The evaluation of executive functions unveiled significant inter‐group variances. However, a universally diminished executive performance score across all participants, including the control group, was observed, likely attributable to an amalgamation of risk factors such as malnutrition and unmanaged chronic conditions like diabetes and hypertension.

**Conclusion:**

This research strives to generate prevalence data on cognitive impairment and dementia among Chiclayo’s elderly, mirroring the area’s socio‐economic diversity. By spotlighting the challenges of pathological aging and prevalent risk factors, the study underscores the critical need for heightened public awareness and proactive brain health maintenance to thwart dementia onset.

